# Beating Total Arch Replacement for Aortoesophageal Fistula Involving the Aortic Arch

**DOI:** 10.1016/j.atssr.2023.07.004

**Published:** 2023-08-06

**Authors:** Ryota Matsumoto, Kazuo Shimamura, Takayuki Shijo, Koichi Maeda, Kizuku Yamashita, Toru Ide, Shigeru Miyagawa

**Affiliations:** 1 Department of Cardiovascular Surgery, Osaka University Graduate School of Medicine, Osaka, Japan; 2 Department of Minimally Invasive Cardiovascular Medicine, Osaka University Graduate School of Medicine, Osaka, Japan

## Abstract

Aortoesophageal fistula is a challenging condition, especially when the aortic arch is involved. Optimal treatment, including surgical approach and adjunctive procedures for organ protection, remains debatable. We describe our strategy for treating aortoesophageal fistula involving the aortic arch using total arch replacement with the heart beating (“beating total arch replacement”) under partial cardiopulmonary bypass.

Aortoesophageal fistula (AEF) is a complex condition involving the aorta and esophagus; it is lethal if it is treated conservatively. Both aortic and esophageal repair is essential; however, operative outcomes remain poor,^1^^,^^2^ especially when AEF involves the aortic arch. Aortic arch repair is more complex and challenging because it requires extensive repair and adjunctive techniques for organ protection.^3^^,^^4^ However, excessive procedures may influence recovery by delaying subsequent procedures, such as esophageal repair. To complete the treatment course of AEF, a meticulous treatment strategy to control procedural invasiveness is essential.

We describe our AEF treatment strategy involving the aortic arch using total arch replacement (TAR) with the heart beating (“beating TAR”) under partial cardiopulmonary bypass (CPB).

## Technique

Our strategy for AEF is a radical treatment consisting of both aortic and esophageal repair. In principle, esophagectomy is performed before the radical aortic repair if there are no concerns of bleeding (ie, the aneurysm is excluded by thoracic endovascular aortic repair [TEVAR]). In cases of staged esophagectomy, video-assisted thoracic surgery from the right thoracic cavity is the chosen treatment. Esophageal reconstruction is postponed until the general and nutritional conditions of the patient are improved.

**Figure 1:**
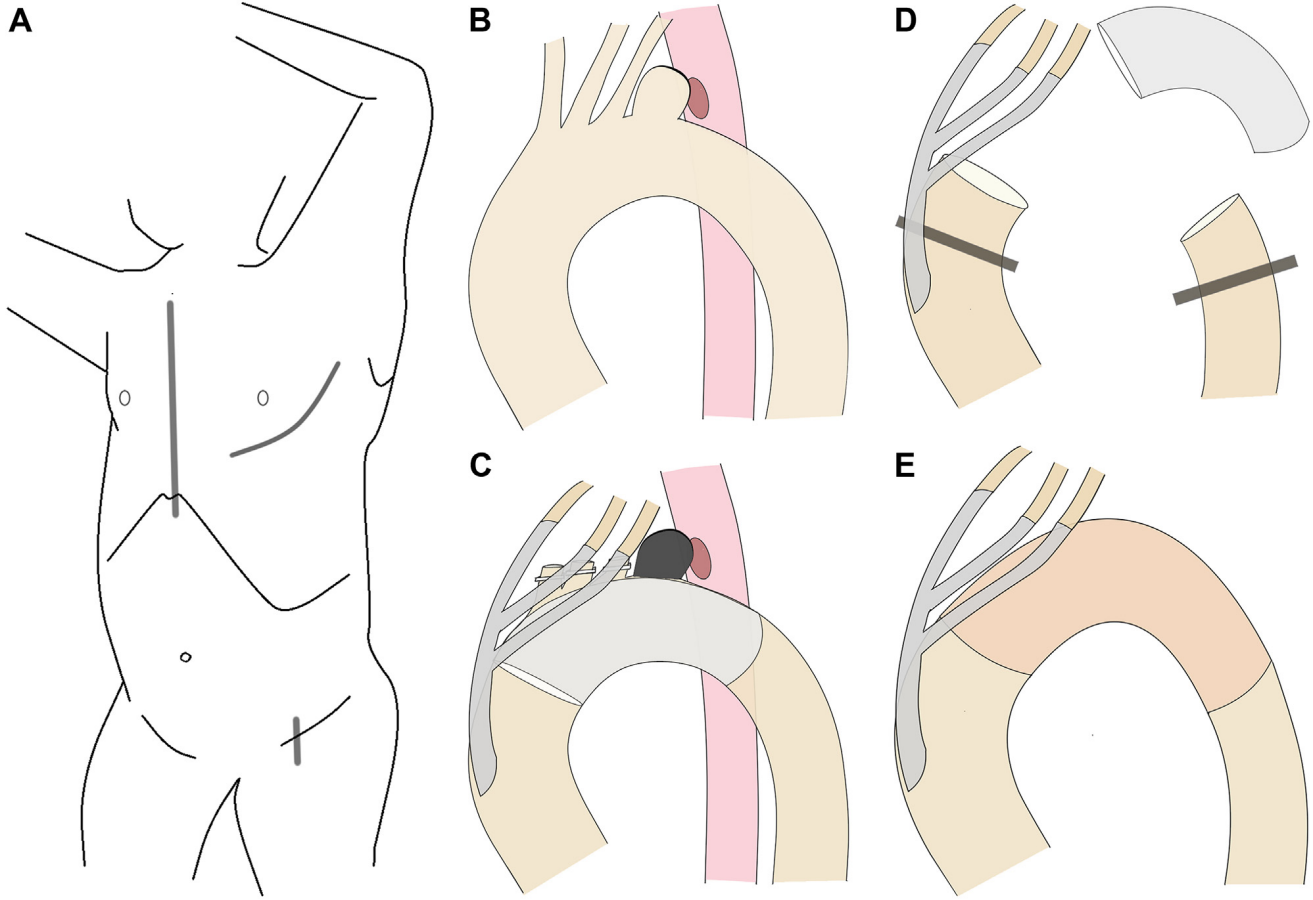
Schematic presentation of surgical technique. (A) Operative position and incisions. (B) Preoperative conditions. (C) Zone 0 thoracic endovascular aortic repair with translocation of the supra-aortic vessels. (D) Aortic clamping and opening of the aortic arch with endograft explantation. (E) Aortic arch replacement with a rifampicin-bonded Gelweave graft.

The patient is intubated with a separate endotracheal tube and placed in the right semilateral decubitus position with the left arm extended over the head. Separate incisions are made for median sternotomy and left anterolateral thoracotomy (Figure 1A). The number of intercostal spaces for thoracotomy depends on the extent of infection. The ascending aorta and supra-aortic vessels are dissected through median sternotomy, whereas the distal aortic arch and descending aorta are dissected through left thoracotomy.

The supra-aortic vessels are translocated proximally before initiation of CPB to make space for clamping of the proximal aortic arch. After systemic administration of 300 U/kg of heparin, the ascending aorta is partially clamped and anastomosed with the inflow branch of a Hemashield trifurcated graft (Maquet Cardiovascular) in a side-to-end fashion. The outflow branches of the trifurcated graft are subsequently anastomosed to the supra-aortic vessels in an end-to-end fashion.

CPB is established by retrograde perfusion from the femoral artery and venous drainage through right atrial or femoral venous cannulation. The body temperature is cooled to 34 °C (tepid hypothermia). After CPB is initiated, the ascending aorta distal to the anastomosis of the inflow graft and the descending thoracic aorta are clamped. Venous drainage and femoral perfusion are carefully regulated by monitoring the radial and femoral arterial pressures; the target systolic pressure of the radial artery is 100 to 120 mm Hg, and the target mean pressure of the femoral artery is 60 to 80 mm Hg.

Once the hemodynamics are stabilized, the aortic arch is opened, and infected prosthetic grafts are explanted. The aneurysm and surrounding tissues are thoroughly débrided without injuring the nerves. A rifampicin-bonded Gelweave graft (Vascutek Terumo) is anastomosed to the ascending aorta. The graft is tunneled through the remnant of the aortic arch and subsequently anastomosed to the descending aorta. After the patient is weaned from CPB and hemostasis is attained, an omental flap is installed to cover the prosthetic grafts.

**Figure 2:**
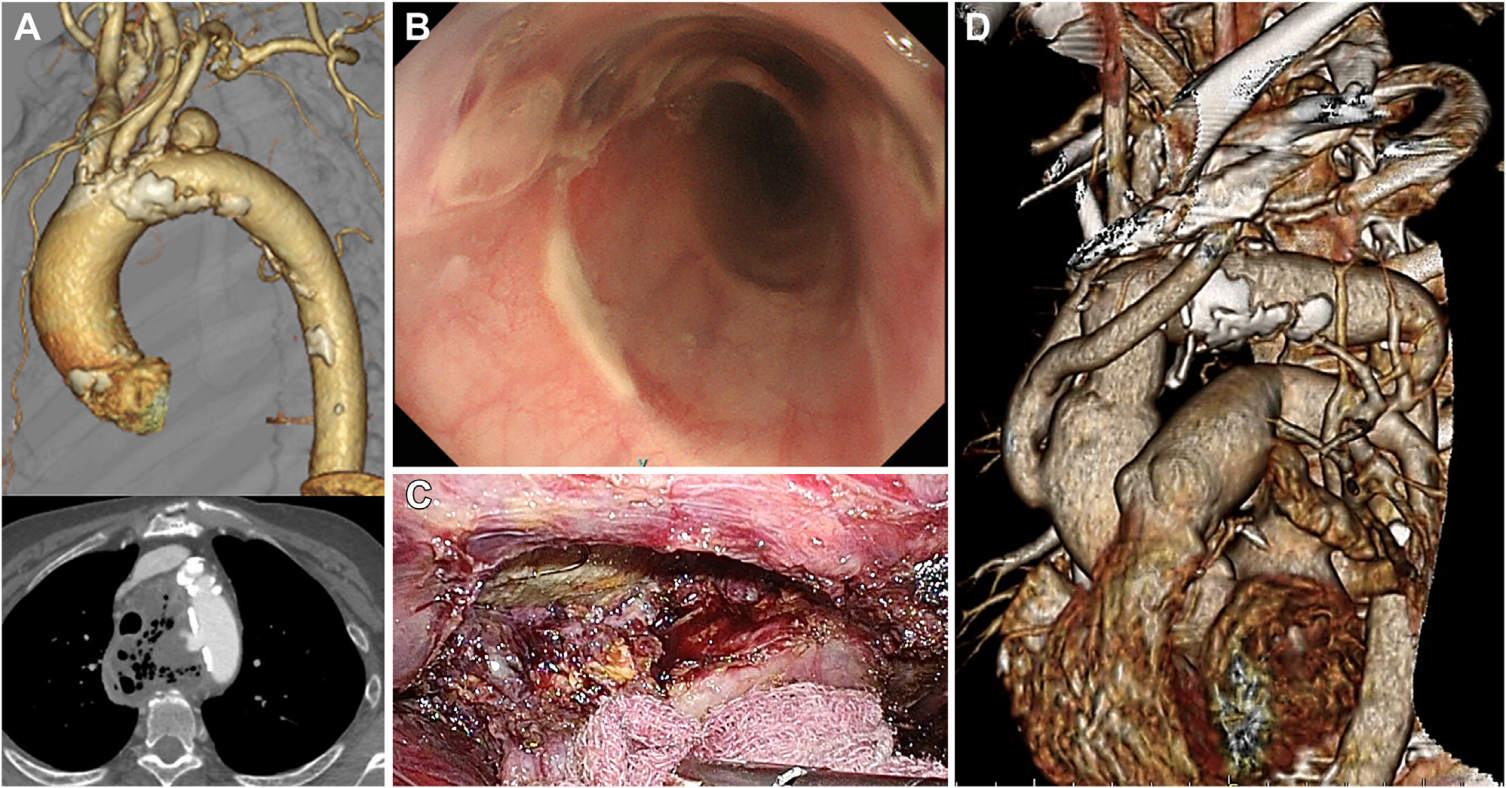
(A) Preoperative computed tomography angiography images. (B) Gastroesophageal endoscopic findings. (C) Intraoperative view during video-assisted esophagectomy. (D) Postoperative computed tomography angiography image.

Using this technique, we treated a 77-year-old woman who presented with fever and fatigue. Computed tomography angiography revealed a saccular aneurysm of the aortic arch compressing the esophagus and trachea, with abscess formation around the aneurysm and no extravasation (Figure 2A). Gastroesophageal endoscopy revealed mucosal erosion but no apparent fistula, implying an ischemic change caused by external compression (Figure 2B). The diagnosis was of a primary AEF with an impending aortic arch aneurysmal rupture (Figure 1B).

We first performed zone 0 TEVAR using CTAG (W. L. Gore & Associates), with translocation of the supra-aortic vessels through median sternotomy to prevent rupture and to prepare for subsequent beating TAR (Figure 1C). Aortography revealed no endoleak. On the second day, video-assisted thoracic esophagectomy was performed safely (Figure 2C). On the third day, beating TAR was performed through median sternotomy and left thoracotomy through the fourth intercostal space. After CPB was initiated with femoral perfusion, the aortic arch was clamped and opened (Figure 1D). The CTAG was easily explanted, and the aortic arch was replaced with a rifampicin-bonded Gelweave graft (Figure 1E). An omental flap was installed through the aortic hiatus to cover the grafts. The operative and CPB times were 323 minutes and 94 minutes, respectively; the blood loss was 1230 mL. Physical therapy was initiated while awaiting esophageal reconstruction.

## Comment

We described our surgical strategy for AEF involving the aortic arch, with emphasis on the aortic arch repair being performed under tepid hypothermia with the heart beating. In aortic arch repair, cardiac arrest and hypothermic circulatory arrest with or without cerebral perfusion are commonly used. Hypothermic circulatory arrest is effective for organ protection but may be invasive for patients with AEF complicated with sepsis or malnutrition. Particularly, hypothermia worsens the hemostatic function. The current method avoids cardiac arrest and hypothermic circulatory arrest, leading to stable perioperative hemodynamics and hemostasis, which allow an earlier recovery and advancement to subsequent treatment.

The current strategy is controversial because of the need for repetitive operations; the technique, involving a 3-stage repair, is not necessarily minimally invasive. However, compared with the 1-stage radical treatment, this strategy is relatively less invasive and has the following merits.

• Bridging TEVAR stabilizes the patient’s condition by ensuring hemostasis of the ruptured aneurysm; this enables esophagectomy under stable conditions without concerns of bleeding. Hemostasis can be improved by maintaining an interval between TEVAR and esophagectomy, instead of performing esophagectomy just after TEVAR. The interval between each procedure can be shortened or extended, depending on the patient’s condition, provided hemostasis and infection control are achieved. If the aneurysm can be treated with zone 1 or zone 2 TEVAR, simultaneous esophagectomy can be considered.
• In the 1-stage radical treatment, radical aortic repair and esophagectomy are performed simultaneously in the same operating field, such as through left thoracotomy (as reported previously).^3^^,^^4^ In this approach, esophagectomy must be performed after the aortic arch and descending aorta are resected but before they are reconstructed during CPB and hypothermic circulatory arrest if the aorta cannot be clamped. This would lead to longer CPB and circulatory arrest times and more bleeding because various procedures (including esophagectomy) have to be performed under intense heparinization and hypothermia.
• Esophagectomy performed before the radical aortic repair allows implantation of a prosthetic graft in a sterile operating field (not contaminated with AEF), leading to better infection control.

Radical aortic replacement with esophagectomy is the most durable and desirable treatment of AEF. TEVAR (with or without esophagectomy) alone is a palliative treatment. TEVAR with esophagectomy can be performed if the patient is too ill to tolerate the radical treatment; this was not the case for our patient.
